# Medicines reconciliation at the community mental health team–general practice interface: quality improvement study

**DOI:** 10.1192/bjb.2019.42

**Published:** 2020-02

**Authors:** Chris F. Johnson, Karen Liddell, Claudio Guerri, Paul Findlay, Alex Thom

**Affiliations:** 1 NHS Greater Glasgow and Clyde; 2 Anchor Mill Medical Practice, Paisley

**Keywords:** Patient safety, quality improvement, psychiatry, general practice, prescriptions

## Abstract

**Aims and method:**

To increase the proportion of patients with no psychotropic drug discrepancies at the community mental health team (CMHT)–general practice interface. Three CMHTs participated. Over a 14 month period, quality improvement methodologies were used: individual patient-level feedback to patient's prescribers, run charts and meetings with CMHTs.

**Results:**

One CMHT improved medicines reconciliation accuracy and demonstrated significant reductions in prescribing discrepancies. One in three (119/356) patients had ≥1 discrepancy involving 20% (166/847) of all prescribed psychotropics. Discrepancies were graded as: ‘fatal’ (0%), ‘serious’ (17%) and ‘negligible/minor harm’ (83%) but were associated with extra avoidable prescribing costs. For medicines routinely supplied by secondary care, 68% were not recorded in general practice electronic prescribing systems.

**Clinical implications:**

Improvements in medicines reconciliation accuracy were achieved for one CMHT. This may have been partly owing to a multidisciplinary team approach to sharing and addressing prescribing discrepancies. Improving prescribing accuracy may help to reduce avoidable drug-related harms to patients.

Drug-related harms contribute to avoidable morbidity, hospital admissions and death.^1–3^ A recent UK report estimates that avoidable drug-related harms due to prescribing errors in the National Health Service (NHS) in England cost £98.5 million annually, consume 181 626 hospital bed days, contribute to 1708 deaths and cause 712 deaths.^3^ As more than 1.2 billion NHS prescriptions are dispensed in the UK and Northern Ireland each year,^4,5^ and the majority of people receive care in the community, it is not surprising that the majority of the estimated costs (£83.7 million) and deaths (627) are associated with primary care.^3^ However, some of these drug-related harms are associated with potentially avoidable prescribing errors.^3^ Prescribing errors occur when ‘as a result of a prescribing decision or prescription writing process, there is an unintentional significant reduction in the probability of treatment being timely and effective or increase in the risk of harm when compared with generally accepted practice’.^6^

Medicines reconciliation at healthcare interfaces can help to minimise prescribing errors and drug-related risks to patients, reducing hospital admissions and accident and emergency visits.^7^ Medicines reconciliation is the systematic process of identifying an accurate list of a person's current medicines and comparing them with the current list in use, recognising any discrepancies and documenting any changes, resulting in a complete list of medicines.^8^ However, the majority of medicines reconciliation studies and guidance have focused on in-patient and secondary care hospitals at the point of admission or discharge; these are lacking for primary care and non-acute settings.^7–12^

People attending community mental health teams (CMHTs), with or without serious mental illness, experience more multimorbidity and polypharmacy,^13^ receive multiple psychotropics^14^ and high-risk medicines,^12,15^ and experience varying degrees of cognitive impairment, disorganised thinking and impaired insight into their conditions due to mental illness.^16^ All of this may contribute to potentially avoidable drug-related harms, thereby placing greater responsibility on clinical staff to ensure accurate prescribing. Finally, previous CMHT–general practice audits have demonstrated that up to 42% of CMHT attendees had ≥1 psychotropic prescribing discrepancy.^17^ This study aims to improve psychotropic prescription reconciliation accuracy at the CMHT–general practice interface.

## Aims and objectives

To increase the proportion of CMHT patients who have their psychotropic prescriptions accurately reconciled and recorded within their regular CMHT review letters to ≥80% by January 2017.

## Method

### Design and setting

Quality improvement methodologies were used from October 2015 to January 2017. The UK's NHS is taxpayer funded and devolved in the home nations. NHS Greater Glasgow & Clyde (NHSGGC) provides healthcare services for a diverse population of approximately 1.2 million people across a varied urban area containing 241 general practices and 18 CMHTs with more than 18 000 patients attending annually. CMHTs support and/or treat people with mental health illness and/or difficulties in out-patient and domiciliary settings, providing more than simply out-patient psychiatric treatment. Three CMHTs were approached and agreed to participate.

### Ethics

Approval was sought from the West of Scotland Ethics Service; however, as the work was considered to be service improvement and evaluation primarily undertaken to support prescribers and to optimise normal patient care, ethical approval was not required.

### Patient inclusion

All patients were eligible for inclusion if they attended the CMHT for review by a psychiatrist or junior doctor in the 4 weeks prior to data collection. Patients were identified from each CMHT's appointment systems, i.e. computer systems and clinic sheets, and ranked by attendance date from most to least recent. Systematic random sampling was applied with every *n*th patient being included, e.g. 100 attendees identified, and every 10th patient was sampled, giving 10 patients per reconciliation cycle per CMHT. Clinical notes for selected patients were then accessed by one clinical pharmacist (C.J.) to obtain the most recent CMHT clinic letter to the patient's general practitioner (GP). Where clinical notes were not available, the next patient was included, e.g. if the 10th patient's notes were not available, the 11th patient was included, and so on. Any patients who had been included in a previous medicines reconciliation cycle were excluded from subsequent cycles, as they did not represent routine care.

### Data collection

We planned to have 12 medicines reconciliation cycles, every 8 weeks for 24 months starting from October 2015, with a single data collector (C.J.). However, the support of a pharmacy technician (C.G.) was secured, which enabled more frequent 4-weekly data collection from June 2016 to January 2017. The technician was trained in the use of a standardised data collection form, specifically piloted and tested for medicines reconciliation, before undertaking this work.

The standardised data collection form was used to collect patient-level information from the most recent CMHT letter: patient name, age, gender, address, residential postcode to allow mapping of Scottish Index of Multiple Deprivation (SIMD) codes,^18^ Community Health Index (CHI) number, psychiatrist's name, psychiatric diagnoses (classified according to ICD-10^19^ with primary diagnosis being used in the analysis), psychotropic prescribing information (drug, form, dose, dose instructions and indication), GP's name, and general practice name and address.

### Medicines reconciliation

General practices were then contacted to arrange a suitable time to access and review patients' records. The general practice electronic record was considered to represent the most accurate prescription list, as it contains psychotropic and non-psychotropic prescription information for medicines initiated and continued by GPs and specialists, and supplied for patients by GPs on NHS prescriptions, and also populates the Emergency Care Summary (ECS). ECS allows authorised clinicians to access general practice prescribing information in different healthcare settings and interfaces.^20^ CMHT prescribing information was then reconciled against the patient's general practice records (EMIS or Vision and Docman) by a clinical pharmacist (C.J.) or pharmacy technician (C.G.). Where discrepancies were identified, these were recorded. A medicines discrepancy was defined as any intentional or unintentional difference, including but not limited to omission, addition or mismatch of drug, dose, dose instructions, preparation and/or route of administration for psychotropic medicines between a patient's most recent CMHT letter and general practice prescribing records.^8^

General practice prescription lists were also assessed where appropriate for ‘out of practice medicines’. These are routinely prescribed and supplied by secondary care, i.e. clozapine and antipsychotic depots. Although not mandatory, NHSGGC recommends that ‘out of practice medicines’ are added to electronic records to ensure current prescribed medicines are available on ECS.

As non-psychotropic medicines can influence an individual's mental health and interact with psychotropic medicines, non-psychotropic prescribing information was also collected along with the individual's known drug allergies to complete the patient's current medicines list.

### Intervention

**Fig. 1:**
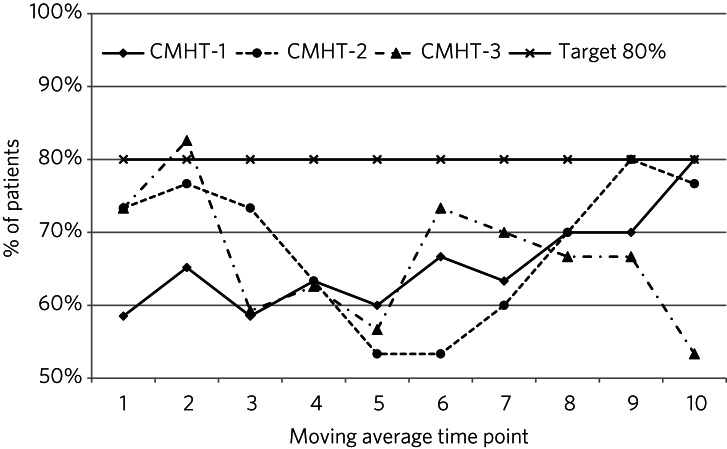
Percentage of patients with no psychotropic medicines reconciliation discrepancies per community mental health team (CMHT) (moving average time point).

The planned quality improvement intervention comprised three parts: (a) individualised prescriber patient-level feedback summaries after each reconciliation cycle; (b) run charts demonstrating the proportion of patients with ≥1 psychotropic medicine discrepancy, as per Fig. 1, starting after the first three reconciliation cycles (‘quarter 1’) were complete, then after each reconciliation cycle; and (c) a planned face-to-face meeting with each CMHT to discuss and reflect on progress. We were aware from previous NHSGGC CMHT–general practice medicines reconciliation work that 58% of patients had no psychotropic prescribing discrepancies;^17^ therefore, ≥80% was considered and set as an appropriate achievable target.

The individualised prescriber patient-level summaries were fed back to the patient's psychiatrist and GP. Feedback was standardised and included date of medicines reconciliation, patient's name and address, CHI, psychiatric diagnosis, complete medicines list (psychotropic and non-psychotropic) and medicines reconciliation summary, i.e. no discrepancies or listing psychotropic discrepancies for the psychiatrist consideration and amendment when the patient was next reviewed or sooner if appropriate. Where non-psychotropic prescribing issues were identified, these were highlighted to the practice's prescribing support pharmacist and patient's GP for consideration and appropriate action.

Although face-to-face meetings with each CMHT were planned, CMHT-1 asked that the quality improvement work be discussed at their multidisciplinary training day that all psychiatrists, junior doctors, community psychiatric nurses (CPNs) and administration staff attend. Meetings with CMHT-2 and 3 each involved two psychiatrists and one senior CPN, as resource limitations and workloads prevented the wider multidisciplinary team from participating.

### Grading of discrepancies

Discrepancies were graded for severity of potential harm to patients, individually by four clinicians with mental health experience (two clinical pharmacists (C.J. and K.L.), a consultant psychiatrist (A.T.) and a GP (P.F.)). We used a grading system modified from a previous in-patient mental health study:^21^ 1, negligible (doubtful or negligible importance); 2, minor (minor adverse effects or worsening of condition); 3, serious (serious adverse effects or relapse); and 4, fatal. Where there was disagreement regarding the severity grading for potential harm to patients, this was resolved by discussion until agreement was reached.

### Analysis

The primary measure of interest was the proportion of patients with ≥1 psychotropic medicines discrepancies. However, to further evaluate the quality improvement work, anonymised patient-level data were then analysed and discrepancy rates per patient were calculated. Owing to the small CMHT samples (10 patients per reconciliation cycle), there was significant variance; therefore, moving averages were calculated and graphed. In addition, owing to the small sample size for each reconciliation cycle per CMHT, and small data cells containing data counts <5, further analysis used aggregated data from the 12 reconciliation time points which were defined as ‘quarters’: quarter 1 = cycles 1–3, quarter 2 = cycles 4–6, and so on.

Data were collated using Excel® and further analysed in SPSS (version 23). Discrepancy rates per quarter were assessed using chi-squared tests. Interrater agreement for discrepancy severity gradings were assessed using Kendall's coefficient of concordance for the four raters.^22^

## Results

Initially, 360 patients were identified and included. However, four patients were excluded: two because one general practice declined to participate, one who died, and one because their GP considered them inappropriate for inclusion. The remaining 356 patients (Table 1) attended 77 general practices, and 33% (119/356) had ≥1 psychotropic medicine discrepancy during the study period.

**Table 1:** Patient demographics by community mental health team

	CMHT-1 *n* = 117	CMHT-2 *n* = 120	CMHT-3 *n* = 119	Total *n* = 356	
Mean age ± s.d. (range), years^a^	47 ± 13.0 (18–71)	44 ± 12.3 (18–68)	48 ± 11.4 (18–68)	46 ± 12.3 (18–71)	
Female (%)	66 (56)	53 (44)	61 (51)	180 (50)	χ^2^ *P* = 0.166
Deprivation (%)					
SIMD quintile 1 (least deprived)	8 (7)	9 (8)	0 (0)	17 (5)	
SIMD quintile 2	19 (16)	16 (13)	5 (4)	40 (11)	
SIMD quintiles 3 and 4^b^	53 (45)	34 (28)	11 (10)	98 (27)	
SIMD quintile 5 (most deprived)	37 (32)	60 (50)	103 (86)	200 (56)	χ^2^ *P* < 0.001
Primary psychiatric diagnosis (%)					
Mood (affective) disorders (F30–39)	37 (32)	34 (28)	49 (41)	120 (34)	
Schizophrenia, schizotypal and delusional disorders (F20–29)	34 (29)	38 (32)	34 (29)	106 (30)	
Neurotic, stress-related and somatoform disorders (F40–48)	28 (24)	13 (11)	11 (9)	52 (15)	
Disorders of adult personality and behaviour (F60–69)	5 (4)	14 (12)	12 (10)	31 (9)	
Other diagnosis and diagnosis under review	13 (11)	21 (18)	13 (11)	47 (13)	χ^2^ *P* = 0.013
Number of psychiatric morbidities identified per patient					
1	93 (80)	101 (84)	84 (71)	278 (78)	
≥2	24 (20)	19 (16)	35 (29)	78 (22)	χ^2^ *P* = 0.036
Number of non-psychiatric conditions being treated^c^					
0	35 (30)	51 (43)	24 (20)	110 (31)	
1	18 (15)	20 (17)	27 (23)	65 (18)	
2	21 (18)	12 (10)	25 (21)	58 (16)	
3	20 (17)	13 (11)	17 (14)	50 (14)	
4	8 (7)	8 (7)	9 (8)	25 (7)	
5	5 (4)	6 (5)	8 (7)	19 (5)	
≥6	10 (8)	10 (8)	9 (8)	29 (8)	χ^2^ *P* = 0.090
Number of prescribed medicines, median (range)					
Psychotropics	2 (0–6)	2 (0–8)	2 (0–6)	2 (0–8)	*P* = 0.266^d^
Non-mental health	2 (0–22)	1 (0–17)	3 (0–20)	2 (0–22)	
Total prescribed medicines per patient	5 (0–25)	3 (0–19)	5 (0–22)	5 (0–25)	

CMHT, community mental health team; s.d., standard deviation; SIMD, Scottish Index of Multiple Deprivation.

a. Student's *t*-test: CMHT-2 had marginally significantly younger patients than CMHT-1 (*P* = 0.04) and CMHT-3 (*P* = 0.015); no significant age difference between CMHT-1 and CMHT-3.

b. Scottish Index of Multiple Deprivation (SIMD) 3 and 4 were aggregated owing to small cell size. All cells add up to 355 as one patient's postcode was not available.

c. Non-mental health conditions commonly treated for all patients: 30% pain, 19% primary prevention of cardiovascular disease, 15% asthma, 7% type 2 diabetes mellitus.

d. Mann–Whitney U-test.

**Fig. 2:**
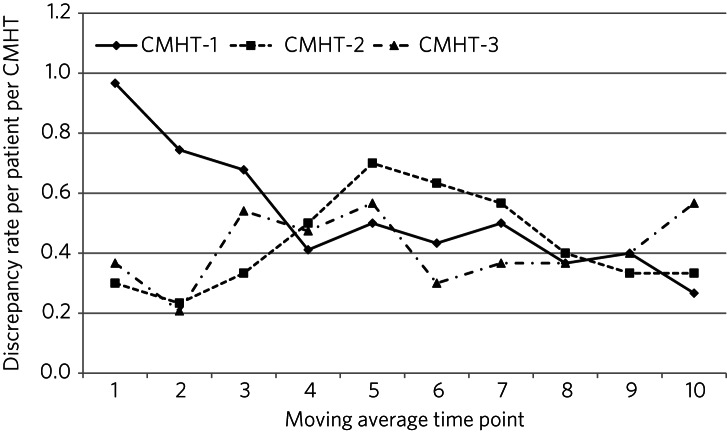
Psychotropic discrepancy rate per patient per community mental health team (CMHT) (moving average time point).

CMHT-1 demonstrated a continuous non-statistically significant improvement in medicines reconciliation accuracy by January 2017 (Fig. 1) and reduction in discrepancy rate per patient (Fig. 2), demonstrating significant reductions in discrepancies by quarter by CMHT (χ^2^ = 13.05, d.f. = 3, *P* = 0.004, Cramer's V = 0.2198). This was not achieved by other CMHTs.

The 356 patients received 847 medicines to treat their psychiatric conditions and adverse drug effects associated with treatment, e.g. clozapine-induced hypersalivation and/or constipation. Of the 847 medicines, antidepressants accounted for 34%, followed by antipsychotics (29%), long-term benzodiazepines and/or z-hypnotics (B-Zs) (15%), other anxiolytics (7%), mood stabilisers (6%), medicines for adverse drug effects (5%) and other medicines (4%) (e.g. methylphenidate, opioid substitution treatment).

Of the CMHT-prescribed medicines, 20% (166/847) were associated with prescribing discrepancies. Of the 166 discrepancies, 43% involved anxiolytics and hypnotics (52 B-Zs, 16 beta-blockers and three pregabalin), 22% antidepressants, 14% antipsychotics, 10% medicines used to treat psychotropic adverse drug effects (procyclidine, laxatives, etc.), 5% mood stabilisers and 3% opioid substitution treatment. All discrepancies were graded for severity for potential harm as follows.

- Negligible (33%): quetiapine 200 mg twice daily, not 400 mg at night as per CMHT letter. Quetiapine modified release tablets prescribed instead of ordinary release tablets, incurring an extra £930 per patient per annum.
- Minor (51%): procyclidine 5 mg three times a day, diazepam 5 mg three times a day, etc., missing from CMHT letter.
- Serious (17%): methadone 90 mg daily missing from CMHT letter, fluoxetine 60 mg daily recorded in CMHT letter but the patient had not ordered it for >18 months.
- Fatal: none identified.

Interrater agreement was fair (Kendall's coefficient 0.55, χ^2^ = 364, *P* < 0.001) prior to consensus being reached.

For the 68% (36/53) of patients receiving ‘out of practice medicines’ (65% (19/29) clozapine and 71% (17/24) antipsychotic depots), these medicines were not recorded in general practice electronic prescribing systems and would not show on ECS.

## Discussion

One CMHT achieved a significant improvement in medicines reconciliation accuracy. This improvement may have been influenced by this CMHT starting as an outlier (Figs 1 and 2), and by their multidisciplinary approach to increasing staff awareness of prescribing discrepancies at their team training event. One in six prescribing discrepancies were graded as having a serious potential risk of harm to patients, with the majority being graded as minor/negligible; however, these were associated with significant avoidable prescribing costs.

### Comparison with literature

This study's finding that 33% of patients had ≥1 prescribing discrepancy is consistent with the recent NHS England report highlighting that prescribing errors in primary care in the UK were comparable to those in the US and EU,^3^ and with the results of a previous acute mental health study including all medicines (psychotropic and non-psychotropic),^23^ but lower than the figure reported for other studies.^10,24^ However, we are cautious in drawing comparisons with the wider literature owing to the majority of studies being in acute in-patient settings and the large variations in measures and methodologies used in previous studies.^7,9^

### Strengths and limitations

The main strengths of this study are as follows. It is the first study, to the authors' knowledge, aimed at prospectively improving medicines reconciliation accuracy at the CMHT–general practice interface through prescriber feedback and reflection using routine individual patient-level data. The inclusion of three CMHTs allowed differences in patient populations and prescribing to be considered in achieving the target, as well as showing that CMHT-1's multidisciplinary team approach to engaging and informing the wider team possibly influenced results, thereby overcoming some of the challenges previously outlined by others.^25^ Another major strength was the prescribing feedback loop, highlighting discrepancies and providing an opportunity for prescribers to see, consider and address discrepancies. Although this study did not set out to identify new prescribing risks, it did identify that ‘out of practice medicines’ – specifically, clozapine and antipsychotic depots – were not routinely recorded in general practice systems and did not show on patients' ECS. Finally, this study addressed some of the questions raised by others regarding a lack of mental health quality improvement studies.^26^

The lack of pre-intervention data demonstrating routine variance in medicines reconciliation accuracy may be considered as a limitation. However, we were conscious that prescribers change prescribing behaviours when they know they are being monitored.^27^ As this study involved accessing clinical records within the CMHT, it was not possible to blind prescribers to the clinical pharmacist's actions and presence. Some may consider CMHT recruitment to have potentially biased results. However, only one CMHT achieved continuous improvements in medicines reconciliation accuracy during the study period. Another potential limitation was that the data collection was labour intensive and relatively slow, owing to a lack of integrated patient-centred electronic systems, and involved 77 general practices, limiting the sample size. These factors delayed the speed of feedback to prescribers. However, if a large sample had been used, creating more individual patient-level prescriber feedback, this may have created prescriber overloaded and disengagement.^25^ Finally, although this quality improvement study involved three CMHTs in a highly urbanised region, which may limit generalisability, the findings may be of interest to others working in similar urban regions.

### Implications for practice

The main challenge is improving medicines reconciliation accuracy across interfaces. In comparison with the general population, CMHT patients commonly have more multimorbidity and polypharmacy,^13,14^ are more commonly prescribed high risk medicines,^12,15^ and experience cognitive impairment and disorganised thinking due to mental illness.^16^ Ensuring the accuracy of prescribing should be an imperative for the multidisciplinary team and prescribers to minimise avoidable drug-related harms, and to optimise treatment and recovery. Poor adherence to treatment may be an issue for some patients; therefore, up-to-date medicines lists are essential in trying to assess and ascertain which medicines people may or may not be taking in relation to their progress. However, pharmacological treatment is just one factor on the road to recovery and living well with serious mental illness.

The greater use of ECS within the CMHT clinics may have enabled prescribers to overcome some of the communication barriers previously highlighted.^28^ Anecdotally, we are aware of prescribing errors affecting continuity of care when patients are admitted to general medical wards, leading to missed clozapine doses and, consequently, re-titration or double dosing of depots. In part, this may be due to the low proportion of ‘out of practice medicines’ – specifically, clozapine and depot antipsychotics – not being recorded in practices' electronic records which populate ECS. There is no contractual obligation to record these medicines, and there previously were greater risks associated with these medicines being issued and dispensed inappropriately. However, since June 2016, EMIS and Vision systems have been modified to reduce the risk of ‘out of practice medicines’ being issued. Therefore, work to increase the electronic recording of these medicines may help to reduce avoidable errors.

Interestingly, 31% (52/166) of all discrepancies were associated with long-term B-Zs. This may be due to multiple factors: B-Z being initiated during a crisis or admission,^29^ poor communication between primary and secondary care,^28^ fragmented care^30^ and health carer factors,^16^ as well as a lack of structured medicines reconciliation and/or proactive medicines review when patients attend their CMHTs or GP.^31^ Long-term prescribing of B-Zs is also a concern, as they are known to worsen cognitive impairment^32^ and depressive symptoms^33,34^ and reduce the efficacy of some psychological therapies,^35^ and are associated with increased mortality for people with schizophrenia.^36^

### Future research

Studies should consider patients' perspectives on quality improvement work and what effect it has on their experiences and healthcare journey, as well as developing systems which enable patients to contribute to the medicines reconciliation process, such as patient-held records. Finally, economic evaluations should assess the influence of service development work on healthcare systems, healthcare professionals, carers and, most importantly, our patients.

### Summary of findings

In conclusion, improvements in medicines reconciliation accuracy were achieved for one CMHT. This may have been partly owing to the multidisciplinary team approach to sharing and addressing prescribing discrepancies. Improving prescribing accuracy may help to reduce avoidable drug-related harms to patients.

## About the authors

**Chris F. Johnson** is an Advanced Prescribing Support and Specialist Mental Health Pharmacist, in Primary Care at NHS Greater Glasgow and Clyde, Pharmacy Services, Glasgow, Scotland. **Karen Liddell** is an Advanced Prescribing Support Pharmacist and Prescribing Advisor in Primary Care at NHS Greater Glasgow and Clyde, Pharmacy Services, Glasgow, Scotland. **Claudio Guerri** is a Prescribing Support Pharmacy Technician in Primary Care at NHS Greater Glasgow and Clyde, Pharmacy Services, Glasgow, Scotland. **Paul Findlay** is a General Practitioner at Anchor Mill Medical Practice, Paisley, Scotland. **Alex Thom** is a Consultant Psychiatrist at NHS Greater Glasgow and Clyde, Dykebar Hospital, Paisley, Scotland.
